# Preoperative prediction of lymph node metastasis in nonfunctioning pancreatic neuroendocrine tumors from clinical and MRI features: a multicenter study

**DOI:** 10.1186/s13244-022-01301-9

**Published:** 2022-10-08

**Authors:** Hai-bin Zhu, Pei Nie, Liu Jiang, Juan Hu, Xiao-Yan Zhang, Xiao-Ting Li, Ming Lu, Ying-Shi Sun

**Affiliations:** 1 Key Laboratory of Carcinogenesis and Translational Research (Ministry of Education/Beijing), Department of Radiology, Peking University Cancer Hospital and Institute, 52 Fu Cheng Road, Hai Dian District, Beijing, 100142 China; 2 Department of Radiology, Affiliated Hospital of Qingdao University, Shi Nan District, Qingdao, 266000 China; 3 Department of Ultrasonography, Peking University First Hospital, Xi Cheng District, Beijing, 100034 China; 4 Department of Radiology, Peking University First Hospital, Xi Cheng District, Beijing, 100034 China; 5 Department of Radiology, First Affiliated Hospital of Kunming Medical University, Wuhua District, Kunming, 650032 China; 6 Department of GI Oncology, Peking University Cancer Hospital and Institute, 52 Fu Cheng Road, Hai Dian District, Beijing, 100142 China

**Keywords:** Pancreatic neuroendocrine tumors, Lymph node metastasis, Magnetic resonance imaging, Diffusion-weighted imaging, Outcomes

## Abstract

**Background:**

The extent of surgery in nonfunctioning pancreatic neuroendocrine tumors (NF-PNETs) has not well established, partly owing to the dilemma of precise prediction of lymph node metastasis (LNM) preoperatively. This study proposed to develop and validate the value of MRI features for predicting LNM in NF-PNETs.

**Methods:**

A total of 187 patients with NF-PNETs who underwent MR scan and subsequent lymphadenectomy from 4 hospitals were included and divided into training group (*n* = 66, 1 center) and validation group (*n* = 121, 3 centers). The clinical characteristics and qualitative MRI features were collected. Multivariate logistic regression model for predicting LNM in NF-PNETs was constructed using the training group and further tested using validation group.

**Results:**

Nodal metastases were reported in 41 patients (21.9%). Multivariate analysis showed that regular shape of primary tumor (odds ratio [OR], 4.722; *p* = .038) and the short axis of the largest lymph node in the regional area (OR, 1.488; *p* = .002) were independent predictors for LNM in the training group. The area under the receiver operating characteristic curve in the training group and validation group were 0.890 and 0.849, respectively. Disease-free survival was significantly different between model-defined LNM and non-LNM group.

**Conclusions:**

The novel MRI-based model considering regular shape of primary tumor and short axis of largest lymph node in the regional area can accurately predict lymph node metastases preoperatively in NF-PNETs patients, which might facilitate the surgeons’ decision on risk stratification.

**Supplementary Information:**

The online version contains supplementary material available at 10.1186/s13244-022-01301-9.

## Key Points

- This study developed a MRI model for predicting LNM in NF-PNETs.
- The MRI model showed good performance in validation group (AUC 0.849).
- MRI-defined LNM groups showed significantly different disease-free survival.
- This MRI model might facilitate the surgeons’ decision on risk stratification.

## Introduction

Pancreatic neuroendocrine tumors (PNETs), which arise from the pancreatic neuroendocrine cells, account for 1–3% of pancreatic tumors and rank the second most common malignancies of pancreas [1–4]. Nonfunctioning PNETs (NF‑PNETs) are much more common than functioning PNETs, account for approximately 70–90% of all PNETs [5]. NF‑PNETs are associated with heterogeneous biological behaviors ranging from indolent to highly aggressive [6]. The clinical outcomes are variable with the 5-year survival rates ranging from 30 to 66% [7].

Up to now, surgical resection is still the first choice in the treatment guidelines. However, there is no consensus regarding the extent of the surgical approach, partly due to the challenge of preoperatively predicting Lymph node metastasis (LNM), which has been clearly proved as a significant prognostic factor associated with outcomes. For example, The European Neuroendocrine Tumor Society (ENETS) consensus guidelines recommend pancreatectomy with regional lymphadenectomy should be routinely performed for PNETs > 2 cm [8]. In contrast, National Comprehensive Cancer Network (NCCN) guidelines recommend radical surgery with regional lymph node resection for tumors > 2 cm or those with positive lymph nodes, while for tumors of 1–2 cm, lymphadenectomy should be considered [9]. Therefore, accurately preoperative prediction of LNM is essential in surgery decision-making which can avoid overtreatment for low-risk PNETs patients.

Previous studies found many factors such as tumor size, grade, Ki-67 are associated with LNM in PNETs patients [10–15]. However, these variables are mainly based on postoperative pathological results. As a noninvasive imaging modality, magnetic resonance imaging (MRI) plays a crucial role in detecting and staging PNETs as well as evaluating biological behavior of the tumors [16–19]. Anatomic and functional parameters of MRI have been used as predictors for tumor grade, recurrence, and outcomes after surgery [20–22]. However, few studies focused on the performance of MR in predicting LNM of PNETs. The purpose of this study was to evaluate the clinical characteristics and MRI features in the preoperative prediction of LNM and outcome in PNETs patients with a multicenter dataset.

## Materials and methods

### Patients

The multicenter retrospective study was derived from 4 hospitals in China. This study was conducted in accordance with the Declaration of Helsinki and was approved by the institutional review board of Peking University Cancer Hospital; the informed consent was waived.

**Fig. 1:**
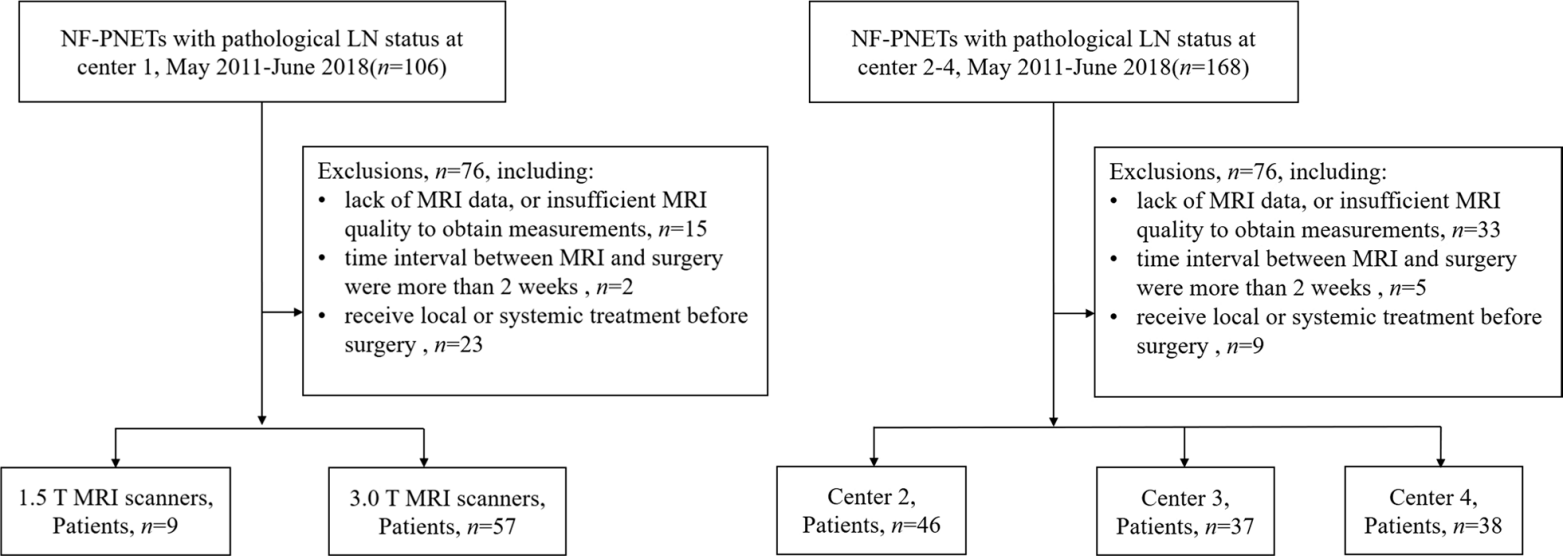
Flowchart of the study of the enrolled patients

The medical records from the 4 hospitals were searched from May 2011 to June 2018. All resected NF‑PNETs with definite pathologically confirmed LN status were enrolled, and the patients were excluded according to the following exclusion criteria: (1) no MRI available or MR images were not sufficient to analysis; (2) the time interval between MRI and surgery was more than two weeks; (3) patient received local or systemic treatment before surgery. The recruitment pathway is shown in Fig. 1. A total of 66 patients from Beijing Cancer Hospital constituted the training group, whereas 121 patients from Peking University First Hospital (*n* = 46), the Affiliated Hospital of Qingdao University (*n* = 37) and First Affiliated Hospital of Kunming Medical University (*n* = 38) constituted the validation group.

Baseline clinical information consisting of gender, age, body mass index (BMI), symptom (present or absent), total bilirubin (TB), alanine aminotransferase (ALT), aspartate aminotransferase (AST), fasting blood glucose (FBG), neutrophil–lymphocyte ratio (NLR = lymphocyte count/ neutrophil count), carcinoembryonic antigen (CEA), carbohydrate antigen 199 (CA199), carbohydrate antigen 724 (CA724), and neuron-specific enolase (NSE) were acquired from the medical records. Pathological analysis was based on the WHO 2019 classification, including the tumor grade (according to mitotic count and Ki-67 index), vascular invasion, neural invasion, and lymph node status.

### MR protocols

All examinations were performed on 1.5 T (*n* = 37) or 3.0 T (*n* = 150) MRI scanners, using an 8-channel phased array body coil with the patients in the supine position. The MRI sequences included T2-weighted single-shot fat spin echo (SSFSE), FSE T1- weighted imaging and DWI. DWI was performed with single-shot echo-planar imaging (EPI) sequence prior to contrast administration with at least with b value of 0 and 1000 s/mm^2^. Dynamic Contrast-enhanced MRI was performed using a breath-hold fat-suppressed 3D T1-weighted LAVA-Flex sequence before and after intravenous administration Gd-DTPA (Magnevist, Bayer Schering Pharma, Berlin, Germany) at a dose of 0.1 mmol/kg and 2 mL/s, followed by a 20 mL of saline solution flush using a power injector. Images were acquired in arterial phase (20–35 s), portal phase (60–80 s), and delayed phase (180–240 s), respectively. The MR protocols are listed in Additional file 1: Table S1.

### MRI features analysis

All the patients were distributed in random order, and the reviewers were blinded to the clinical information and the pathological reports.

#### Qualitative analysis

**Fig. 2:**
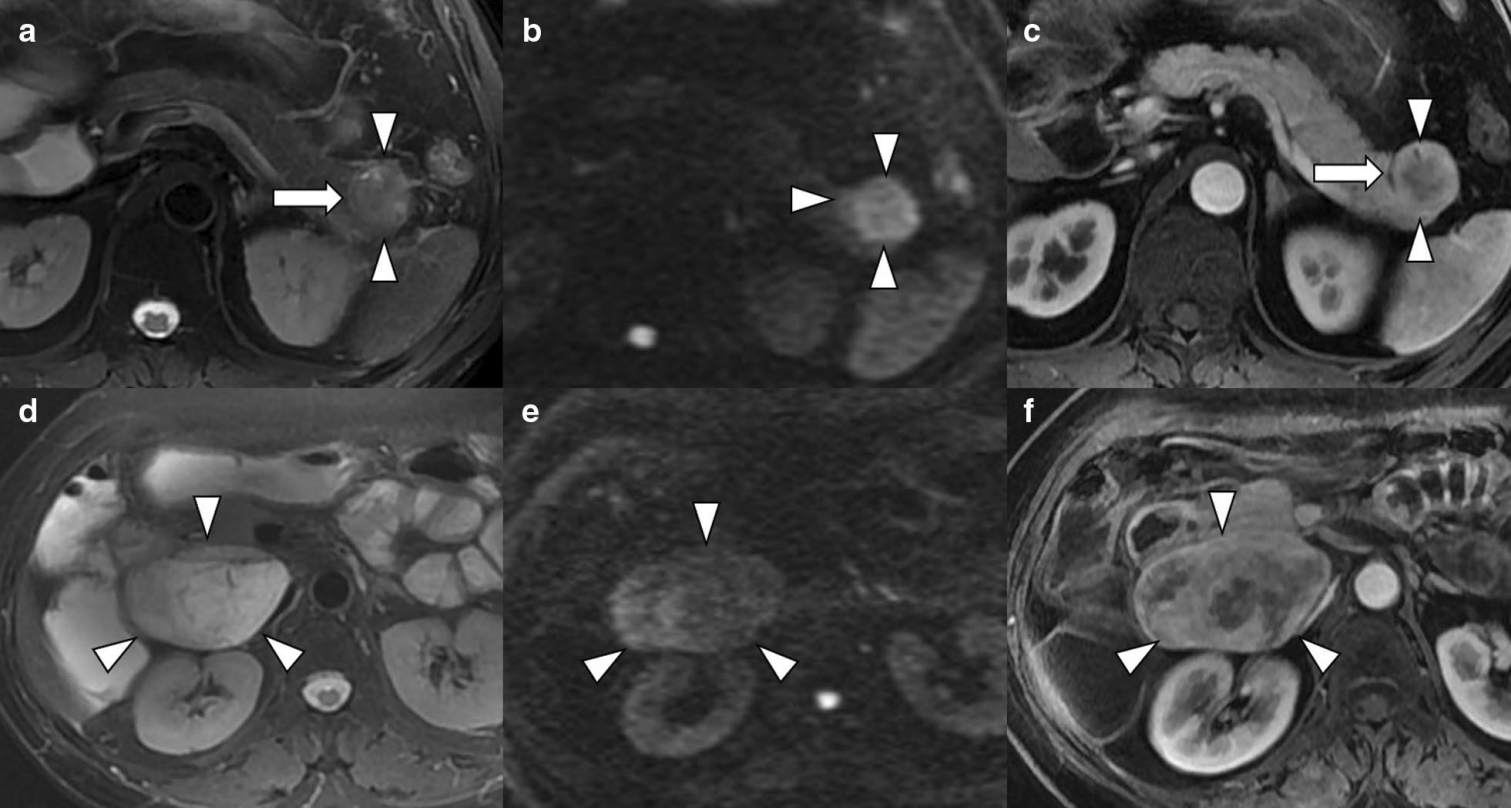
Pancreatic neuroendocrine tumors with regular tumor characteristics on T2WI (left), DWI (middle) and arterial phase (right) images. **a**–**c** a round, well-demarcated tumor with smooth contours is shown on MR images. The tumor-pancreas interface is sharp with pseudo-capsule (arrow). **d**–**f** MR images reveal oval but regular tumor located at the head of pancreas, the tumor-pancreas interface is clear and smooth. Although the pseudo-capsule is not demonstrated on MR images, the tumor is also categorized with regular shape

**Fig. 3:**
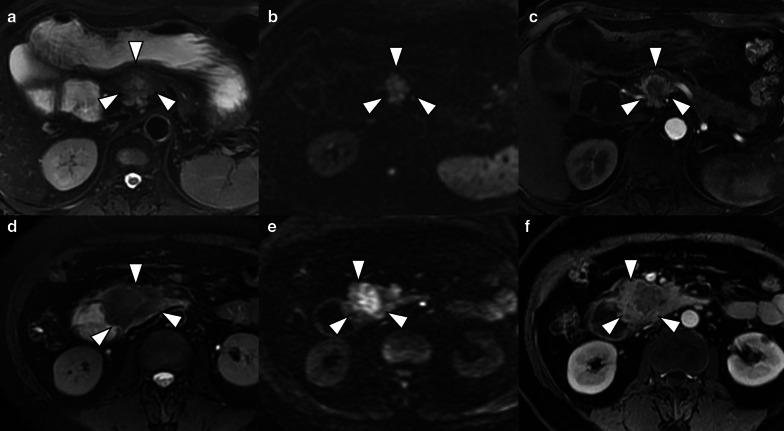
NF-PNETs reveal irregular characteristics on T2WI (left), DWI (middle) and arterial phase (right) images. The tumor shows ill-defined nodular tumor–pancreas interface with infiltrative to adjacent normal pancreatic parenchyma on (**a**–**c**). Highly infiltrative tumor with confluent multinodular growth and lacking demarcation is detected on (**d**–**f**), showing directly penetration to the duodenum with pathologic proven

Two radiologists evaluated the qualitative variables independently (H.B.Z. and P.N., both with 12 years' experience in abdominal MRI), and inter-observer agreement was evaluated. When there was a discrepancy, a senior radiologist (X.Y.Z., with 15 years' experience in abdominal MRI) was introduced for arbitration, and the result after arbitration was used in next analysis. The following qualitative features were evaluated: (a) tumor location (pancreatic head/neck, body or tail), (b) size (maximal axial dimension), (c) signal intensity (SI) on T2-WI (hypointense, isointense, or hyperintense relative to the surrounding pancreatic parenchyma), (d) exophytic growth (present or absent), (e) hyperenhancement at arterial phase (present or absent), (f) presence of upstream common bile duct dilatation (CBDD) and/or main pancreatic ductal dilatation (MPDD) due to tumor compression, (g) presence of vascular and adjacent organs invasion, (h) presence of synchronous liver metastases, (i) tumor margin (regular or irregular). The distal main pancreatic duct of the tumor was considered dilated when its diameter was ≥ 5 mm, while common bile duct dilatation was defined when its diameter was ≥ 10 mm. Vascular invasion was defined as the tumor directly invaded adjacent vessels with the results of lumen obstruction or occlusion, abutted more than 90° of major peripancreatic arteries, or abutted more than 180° of the adjacent vein. Regular margin was defined as: the round or oval shape with clear demarcation (Fig. 2a–f). Otherwise, the tumor with extra-nodular growth and confluent multinodular growth were defined as irregular margin (Fig. 3a–f) [23, 24]. The interobserver level of agreement for tumor margin was assessed by two blinded radiologists independently.

#### Lymph node assessment

If the tumor was located in the pancreatic head/neck, regional nodes included those along the common bile duct, common hepatic artery, portal vein, the anterior and posterior surfaces of the pancreatic head, and along the superior mesenteric artery. If the tumor was located in the pancreatic body/tail, regional nodes included those along the common hepatic artery, splenic and superior mesenteric artery [25]. All visible regional lymph nodes in the field of scan were analyzed. The size of the largest lymph node (the long axis and short axis) was measured, and short/long ratio was calculated subsequently. The number of the lymph nodes with the short axis > 5 mm, > 10 mm detected on DWI sequence was also recorded. In addition, morphological involvement of LNM was reported when the lymph node with abnormal round morphology or central necrosis.

#### Quantitative analysis

Regions of interests (ROIs) were manually placed on the DWI images with *b* value of 1000 s/mm^2^ by two radiologists working together. DCE-MRI and T2WI images were used as reference for ROI segmentation. ROIs were also drawn long the outer border of primary pancreatic tumor on every slice with carefully avoiding vascular structures, biliary duct, pancreatic duct and normal pancreatic tissue. ADC values from whole slices of the lesion were averaged as the ADC_mean_. The maximum (ADC_max_) and minimum (ADC_min_) ADC value of the tumor were also recorded. Tumor volume was then multiplied by the slice thickness.

### Follow-up after surgical resection

Routine examinations, including radiography and laboratory tests, were performed every 3–6 months for the first 2 years and then annually. Disease-free survival (DFS) was defined as the interval between operation and an event (tumor recurrence, death or last negative follow-up). The last date for follow-up was June 27, 2021.

### Statistics

The differences of the clinical factors and MRI features between LNM and non-LNM groups were compared by using independent *t* test or Mann–Whitney test for continuous variables and Chi-square test or Fisher’s exact test for categorical variables. Interobserver agreement was evaluated using Kappa coefficient, 0.0–0.20, 0.21–0.40, 0.41–0.60, 0.61–0.80 and 0.81–1.00 was considered slight, fair, moderate, substantial and perfect agreement. Univariate logistic regression included 33 variables according to LNM status; Bonferroni correction was used for multiple comparison (*p* < 0.05/33 ≈ 0.0015 was considered as statistically significant, *p* < 0.01 was considered as potentially significant for univariate logistic regression). Multivariate logistic regression model was established by substituting potentially significant variables from univariate analysis into equation. Independent factors associated with LNM were tested with odds ratios (OR) calculated and then, used for establishing a multivariate model for predicting LNM in NF-PNETs. The diagnostic performance of the model was evaluated by receiver operator characteristic (ROC) curve with area under ROC (AUC), sensitivity, specificity, positive predictive value (PPV), negative predictive value (NPV) and accuracy calculated in both the training and validation cohorts. The cutoff value was selected using the maximum Youden’s index. Nomogram was yielded for clinical application. DFS curves of model-defined LNM groups and pathology LNM groups in the validation group were compared using Kaplan–Meier method with log-rank estimates. All statistical analyses were performed with IBM SPSS (Version 22.0; IBM Corp., New York, USA), and R package 3.6.2 (R Foundation for Statistical Computing, Vienna, Austria) Two-sided *p*-value < 0.05 was considered as statistically significant.

## Results

### Patient characteristics

The clinical, pathological and MRI features of the 187 patients in the training and test cohorts are shown in Table 1. No significant difference except clinical symptom (*p* = 0.011) and NSE level (*p* = 0.039) was found in NF-PNETs clinical characteristics between the training and validation group. Among the 187 with PNETs, 41 patients (21.9%) had LNM and 146 patients (78.1%) had no LNM.

**Table 1:** Clinical characteristics, MRI features of patients in the training and validation group

Factors	Training group (*n* = 66)	Validation group (*n* = 121)	*p*
Gender (male/female)	29/37	57/64	0.680
Age, year	53.74 ± 12.11	53.03 ± 14.58	0.740
BMI, kg/m^2^	24.53 ± 4.04	24.67 ± 3.78	0.820
Symptom (present or absent)	34/32	77 /44	0.011*
NLR	2.36 ± 1.35	3.02 ± 4.02	0.770
TB (< 21/ ≥ 21), μmol/L	53/11	79/12	0.490
ALT (< 40/ ≥ 40), IU/L	55/9	77/14	0.820
AST (< 40/ ≥ 40), IU/L	57/9	82/10	0.600
FBG (< 6.1/ ≥ 6.1), mmol/L	40/23	43/24	0.940
CEA (< 5/ ≥ 5), ng/ml	54/6	84/2	0.070
CA199 (< 37/ ≥ 37), U/ml	52/8	75/11	0.920
CA724 (< 5.9/ ≥ 5.9), U/ml	45/1	46/4	0.360
NSE (< 16.3/ ≥ 16.3), ng/ml	31/15	41/7	0.039*
Lymph node metastasis (N0/N1)	21/45	20/101	0.016*
Tumor location (head or neck/ body/tail)	34/18/14	45/34/42	0.096
SI on T2WI (hypointense/ isointense/hyperintense)	5/45/16	8/81/32	0.930
Maximum diameter of the tumor (< = 20/ > 20 mm)	18/48	47/74	0.040*
Tumor margin (regular/ irregular)	35/31	75/46	0.290
Exophytic growth (present or absent)	36 /30	63/58	0.600
MPDD or CBDD (present or absent)	23/43	35/86	0.403
Hyperenhancement at arterial phase (present or absent)	34/32	64/57	0.860
Homogeneity	42/24	70/51	0.440
Vascular and adjacent tissue involvement (present or absent)	15/51	16/101	0.100
Synchronous liver metastases (present or absent)	18/48	11/110	0.001*
Long axis of the largest lymph node, mm	10.02 ± 7.37	10.39 ± 6.36	0.570
Short axis of the largest lymph node, mm	6.00 ± 4.74	6.19 ± 4.21	0.710
Ratio of the long/short axis of the largest lymph node	1.77 ± 0.53	1.81 ± 0.51	0.610
Abnormal Shape of the largest lymph node (present or absent)	13/53	14/107	0.130
Number of the lymph nodes with the short axis > 5 mm	1.20 ± 1.38	1.22 ± 1.20	0.64
Number of the lymph nodes with the short axis > 10 mm	0.36 ± 0.83	0.24 ± 0.66	0.41
ADC_mean_(× 10^–3^ mm^2^/s)	1470.66 ± 497.53	1554.21 ± 544.41	0.337
ADC_max_(× 10^–3^ mm^2^/s)	2550.67 ± 1129.81	2110.37 ± 707.05	0.024*
ADC_min_(× 10^–3^ mm^2^/s)	497.08 ± 646.99	661.49 ± 560.33	0.413
Tumor volume, mm^3^	66,313.99 ± 259,492.01	53,698.35 ± 277,487.86	0.007*

*p value ＜0.05 was statistically significant between training and validation group

*LNM*  lymph node metastasis, *BMI*  body mass index, *TB*  total bilirubin, *ALT*  alanine aminotransferase, *AST* aspartate aminotransferase, *FBG*  fasting blood glucose, *NLR*  neutrophil–lymphocyte ratio, *CEA* carcinoembryonic antigen, *CA199*  carbohydrate antigen 199, *CA724*  carbohydrate antigen 724, *NSE*  neuron-specific enolase, *MPDD* main pancreatic duct dilatation, *CBDD* common bile duct dilatation, *ADC* apparent diffusion coefficient

Substantial to perfect interobserver agreement was obtained for qualitative variables, ICC ranged from 0.787 to 0.926.

### Univariate and multivariate analyses of preoperative clinical factors and MRI features with LNM

In the training cohort, the long axis of the largest lymph node (*p* < 0.001), short axis of the largest lymph node (*p* < 0.001), shape of the largest lymph node (*p* < 0.001), number of the lymph nodes with the short axis > 5 mm (*p* < 0.001) and the number of the lymph nodes with the short axis > 10 mm (*p* < 0.001) were statistically different between LN-positive and LN-negative groups. Together with the variables above, AST (*p* = 0.003), tumor margin (*p* = 0.007), MPDD or CBDD (*p* = 0.009) and synchronous liver metastases (*p* = 0.002) were considered potentially associated with LNM by clinicians.

According to the multivariate logistic regression analysis, short axis of the largest lymph node (OR, 1.488; 95% CI, 1.162–1.907, *p* = 0.002) and irregular margin of tumor (OR, 4.722; 95% CI, 1.093–20.404, *p* = 0.038) were independent factors associated with LNM of NF-PNETs. The results are shown in Table 2. A representative case of NF-PNET is shown in Additional file 1: Figure S1 and S2.

**Table 2:** Univariate and multivariate analyses of preoperative clinical and MRI risk factors for LNM in NF -PNETS in the training cohort

Factors	Univariate analysis			Multivariate analysis	
	LNM-negative group (*n* = 45)	LNM-positive group (*n* = 21)	*p*	OR (95%CI)	*p*
Gender (male/female)	18/27	11/10	0.350		
Age, year	52.87 ± 12.19	55.62 ± 12.03	0.390		
BMI, kg/m^2^	24.75 ± 3.89	24.06 ± 4.40	0.520		
Symptom (present or absent)	23/22	11/10	0.920		
NLR (mean ± SD)	2.29 ± 1.39	3.02 ± 4.02	0.510		
TB (< 21/ ≥ 21), μmol/L	40/5	13/6	0.070		
ALT (< 40/ ≥ 40), IU/L	42/3	13/6	0.016		
AST (< 40/ ≥ 40), IU/L	43/2	14/7	0.003		
FBG (< 6.1/ ≥ 6.1), mmol/L	30/14	10/9	0.240		
CEA (< 5/ ≥ 5), ng/ml	36/5	18/1	0.650		
CA199 (< 37/ ≥ 37), U/ml	39/2	13/6	0.010		
CA724 (< 5.9/ ≥ 5.9), U/ml	28/1	17/0	> 0.999		
NSE (< 16.3/ ≥ 16.3), ng/ml	22/9	9/6	0.510		
Tumor location (head or neck/ body/tail)	24/12/9	10/6/5	0.900		
SI on T2WI (hypointense/ isointense/hyperintense)	2/31/12	3/14/4	0.360		
Maximum diameter of the tumor (< = 20/ > 20 mm)	15/30	3/18	0.106		
Tumor margin (regular/ irregular)	29/16	6/15	0.007	4.722(1.093–20.404)	0.038*
Exophytic growth (present or absent)	23/22	13/8	0.410		
MPDD or CBDD (present or absent)	11/34	12/9	0.009		
Hyperenhancement at arterial phase (present or absent)	27/18	7/14	0.043		
Homogeneity (present or absent)	33/12	9/12	0.017		
Vascular and adjacent tissue involvement (present or absent)	6/39	9/12	0.012		
Synchronous liver metastases	7/38	11/10	0.002		
Long axis of the largest lymph node, mm	7.44 ± 6.25	15.52 ± 6.64	< 0.001*		
Short axis of the largest lymph node, mm	4.11 ± 3.26	10.05 ± 4.93	< 0.001*	1.488(1.162–1.907)	0.002*
Ratio of the long axis to the short axis of the largest lymph node	1.87 ± 0.62	1.61 ± 0.31	0.150		
Irregular shape of the largest lymph node (present or absent)	3/42	10/11	< 0.001*		
Number of the lymph nodes with the short axis > 5 mm	0.71 ± 0.87	2.24 ± 1.70	< 0.001*		
Number of the lymph nodes with the short axis > 10 mm	0.09 ± 0.42	0.95 ± 1.16	< 0.001*		
ADC_mean_ (× 10^–3^ mm^2^/s)	1453.85 ± 314.02	1505.07 ± 755.50	0.600		
ADC_max_ (× 10^–3^ mm^2^/s)	2371.39 ± 590.96	2917.76 ± 1754.11	0.350		
ADC_min_ (× 10^–3^ mm^2^/s)	574.80 ± 691.29	337.94 ± 524.81	0.007		
Tumor volume, mm^3^	32,301.35 ± 67,882.65	139,198.20 ± 447,799.03	0.140		

*LNM*  lymph node metastasis, *OR* odds ratio, *BMI*  body mass index, *TB* total bilirubin, *ALT* alanine aminotransferase, *AST*  aspartate aminotransferase, *FBG* fasting blood glucose, *NLR*  neutrophil–lymphocyte ratio, *CEA* carcinoembryonic antigen, *CA199* carbohydrate antigen 199, *CA724* carbohydrate antigen 724, *NSE* neuron-specific enolase, *MPDD* main pancreatic duct dilatation, *CBDD* common bile duct dilatation, *ADC* apparent diffusion coefficient

*Bonferroni correction was used for multiple comparison, *p* < 0.0015 was considered as statistically significant for univariate logistic regression

### Constructed model for predicting LNM in NF-PNETs

**Fig. 4:**
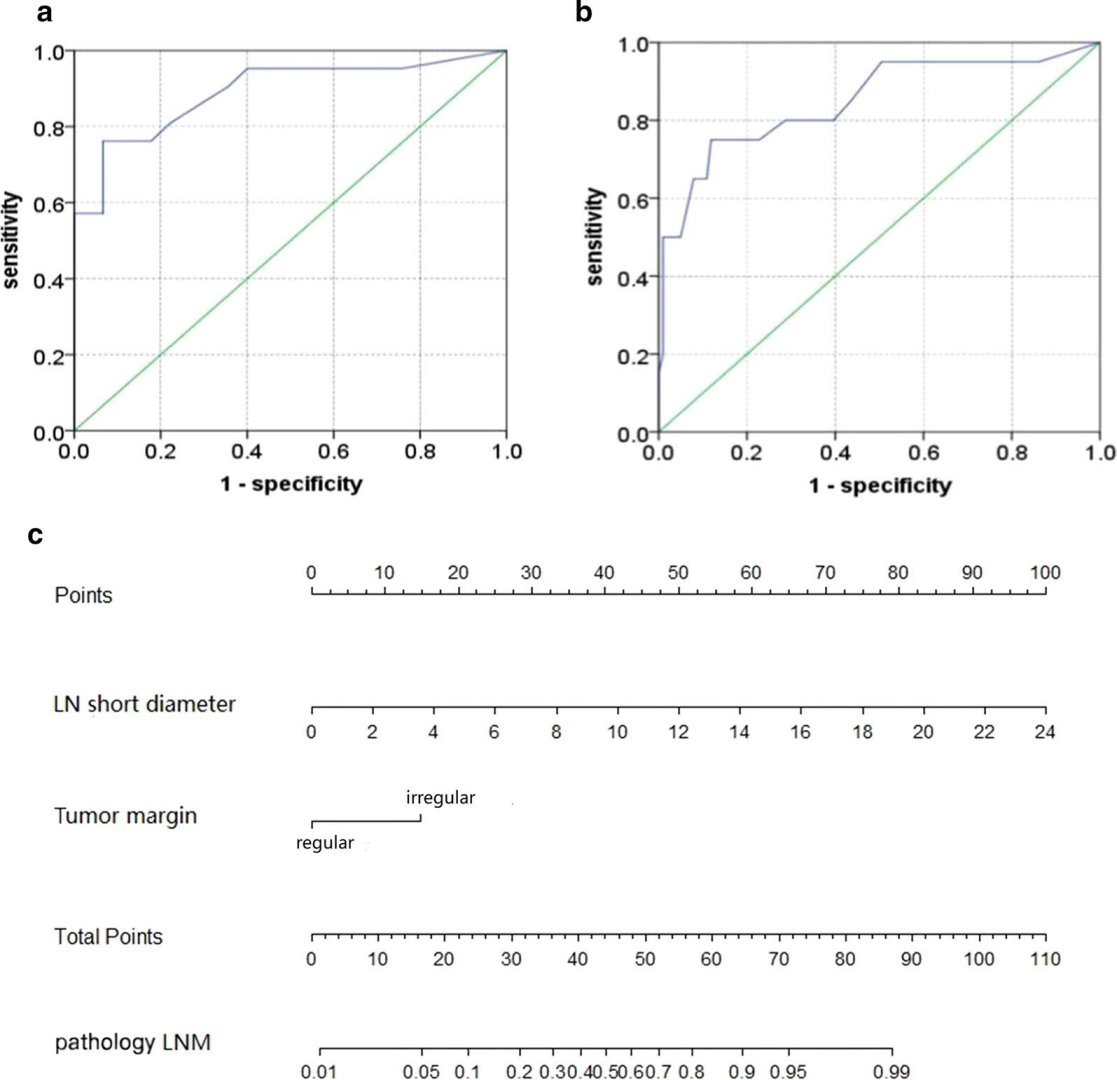
Receiver operating characteristics curves and nomogram. **a**, **b** the receiver operating characteristics curves of the model for predicting of LNM in the learning and validation group. **c** the nomogram yielded from the training cohort for prediction of LNM in NF-PNETs. LNM: lymph node metastasis

A multivariate model for predicting LNM in NF-PNETs was established as *Y* = 0.398* short axis of the largest lymph node (mm) + 1.552* tumor margin (1, regular; 2, irregular) according to the multivariate logistic regression results. The AUCs of the model in the training and validation cohorts were 0.890 (95% CI, 0.795–0.986) and 0.849 (95% CI, 0.740–0.957), respectively (Fig. 4a, b). The cutoff of *Y* was 5.7; *Y* > 5.7 indicated LNM and *Y* ≤ 5.7 indicated non-LNM. The diagnostic performance from parameters is shown in Table 3. The nomogram yielded from the training cohort is shown in Fig. 4c.

**Table 3:** The diagnostic performance of model in predicting LNM in NF-PNETs in the training and validation group

	AUC (95% CI)	Sensitivity (%)	Specificity (%)	PPV (%)	NPV (%)	Accuracy (%)
Training group	0.890 (0.795–0.986)	76.2 (16/21)	93.3 (42/45)	84.2 (16/19)	89.4 (42/47)	87.9 (58/66)
Validation group	0.849 (0.740–0.957)	75.0 (15/20)	88.1 (89/101)	55.6 (15/27)	94.7 (89/94)	86.0 (104/121)

*LNM* lymph node metastasis, *AUC* area under curve, *PPV* positive predictive value, *NPV* negative predictive value

### Relationship between lymph node status and patient outcome

**Fig. 5:**
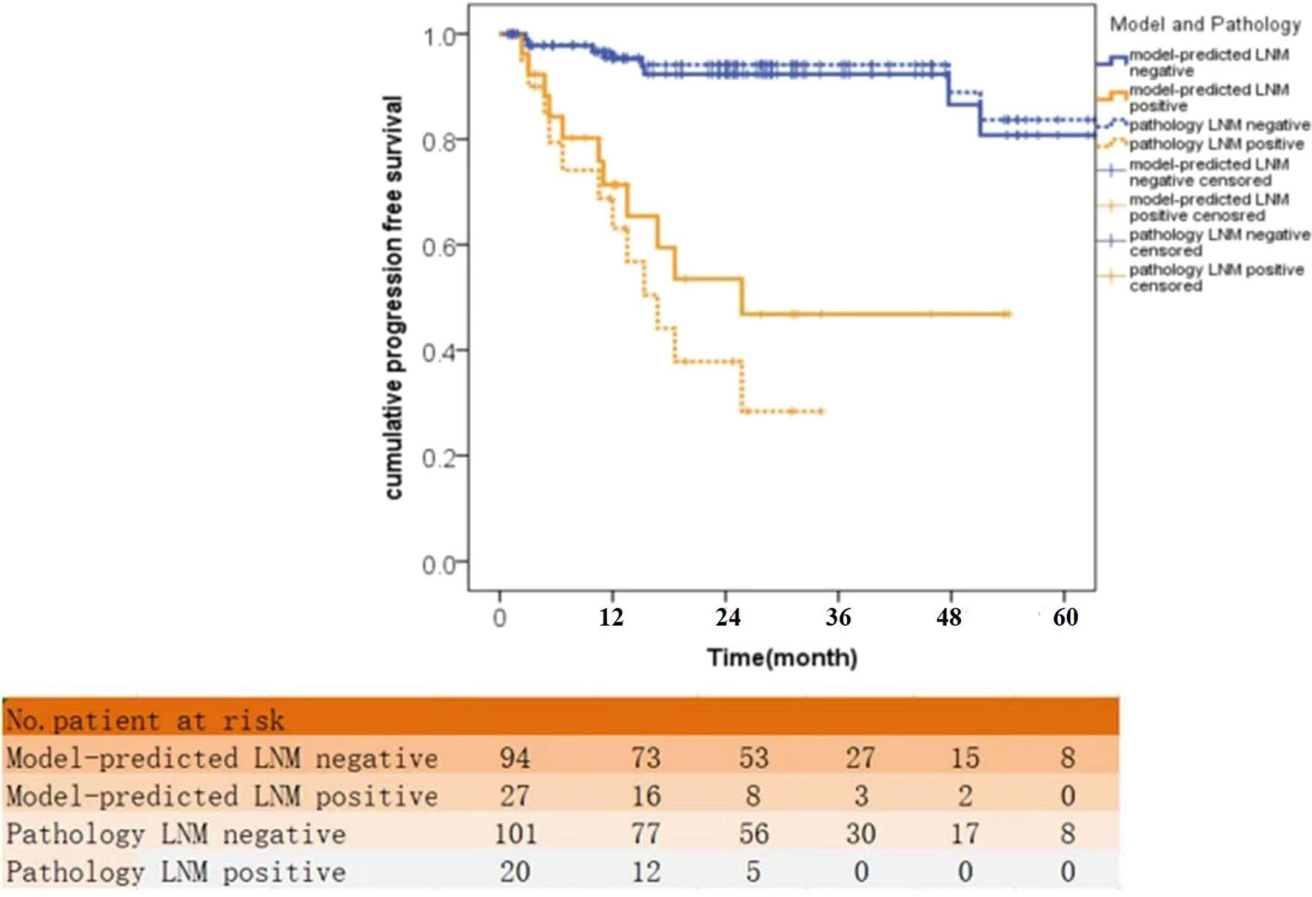
Kaplan–Meier analyses of DFS for the pathological defined LNM patients and the model-defined LNM patients. DFS: disease-free survival, LNM: lymph node metastasis

The median follow-up time of all the patients in the validation group was 24 month (95%CI, 18 to 26 month). The pathological LNM patients demonstrated inferior DFS compared with non-LNM patients (36-month’ DFS rate: 28.4% vs 94.1%, *p* < 0.001). The model-defined LNM patients also demonstrated inferior DFS compared with non-LNM patients (36-month’ DFS rate: 46.8% vs 92.3%, *p* < 0.001). There were neither difference in DFS between pathological LNM and model-defined LNM patients, nor in DFS between pathological and model-defined non-LNM patients (both *p* > 0.05). Survival curves according to the lymph node status are shown in Fig. 5.

## Discussion

Because biological behavior of NF-PNETs tends to be varied across pathological and molecular heterogeneity, the currently used guidelines for management of PNETs have been debatable, especially the concerned whether regional lymph node should be dissected, although essential for patients, remains unsolved. In this multicenter study, we analyzed the relationship between preoperative clinical and MRI features with the LN status from 187 NF-PNETs. We found that irregular margin of the pancreatic tumor and short axis of the largest lymph node were independent predictors for LNM in the multicenter study, achieving an AUC of 0.890 and 0.849 in the training and validation cohorts, respectively. In addition, the MRI features-predicted LNM was associated with DFS, indicating the radiologic factors can be used as a potential biomarker to predict patient outcome, which might guide surgeons’ decision-making before surgery and individualized management of NF-PNETs patients.

Our results showed that NF-PNETs with irregular margin of the tumor appeared to be more infiltrative and metastatic to adjacent lymph nodes, which was consistent with previous studies. Okabe et al. [23] found that ill-defined margin was not only significantly correlated with clinical variables relevant to disease progression such as tumor size, high histologic grade, synchronous liver metastasis and lymph node metastasis, but also independently correlated with poor overall survival. Similarly, De Robertis et al. [26] reported ill-defined margins were more common in G2-3 and stage III–IV PNETs than in G1 and low-stage tumors with high specificity in identifying G2-3 and stage III–IV tumors (90.3% and 96%). Our current data showed that tumors with irregular margins were 4.72 times more likely to have malignant lymph nodes than those with well-defined margins, thus further validating the association between irregular margin and tumor aggressiveness in PNETs. This phenomenon is characterized by the existence of diffuse or local attenuation of enhancement within the ill-defined tumor in comparison with regular shape tumor, which was associated with hypoxia that further induced increase frequency of metastases, high risk of resistance to following treatment and decrease in overall survival.

Short axis of the largest lymph node was also found to be an independent critical predictor for LNM. Previous reports have shown that radiologic lymph node enlargement can be used to differentiate PNETs with or without LNM. Choi et al. [27] investigated the CT features of 166 NF-PNETs to predict LNM and identified radiologic lymph node enlargement as a strong independent predictor for LNM, achieving the highest OR (11.76) as compared with other predictors. Partelli et al. [28] analyzed the clinical, pathological and radiological features of 188 non-functioning PNETs. Radiological nodal status (OR, 5.58) and tumor grade (G2 vs G1: OR, 4.87) were independent predictors for LNM (AUC, 0.80). Although many other quantitative parameters from lymph node, such as LN short/long ratio and the number of LNs which were believed to reflect the metastatic burden and associated with the prognosis in many cancers, were not predominant independent factors in NF-PNETs in this study.

Previous studies confirmed the association between tumor size and LNM with various cutoff values of the tumor size. For example, Tsutsumi et al. [29] identified PNETs with a tumor size of ≥ 1.5 cm as an indication for malignant lymph node. Jiang et al. [30] evaluated the preoperative parameters for predicting LNM in 100 NF-PNETs and found radiological tumor diameter > 2.5 cm could be used as a reliable predictor of LNM. Choi et al. [31] and Partelli et al. [28] found that the optimal threshold of tumor size for LNM should be 2 cm and 4 cm, respectively. In contrast, a large clinical study performed by Jutric et al. [32] and the lymph node status of 2735 PNETs patients were analyzed, which showed that the incidence of LNM was 24% in the grade 1 tumors less than 1 cm. These results indicate the difficulty and unreliability of identifying LNM by assessing tumor size. In our study, we suggested that the tumor size was not an independent risk predictor for LNM at multivariate analysis, which was consistent with the results of previous studies.

Recently, ADC values derived from DWI which, can noninvasively reflect the structural and functional changes in the biological microenvironment in a quantitative manner, have been reported as an imaging marker to recognize aggressiveness and grade of PNETs. However, information on the relationship of ADC values and LN status was still relatively rare. Harimoto et al. [33] found that neural invasion and lower mean ADC values (≤ 1458 × 10^–6^ mm^2^/s) were independent predictors of LNM. We should note that only ADC_min_ was significantly predictive factors for the histological lymph node metastasis in the univariate analysis in our study, which was not an independent risk predictor for LNM in PNETs in the multivariate analysis. Several factors may contribute to the different results. First, the clinical behavior and histopathologic appearances of PNETs varies widely from benign to highly malignant neoplasms, which may further lead to the overlap between the ADC values. Similar studies were also observed in the assessment of DWI for predicting the histologic grade of PNETs. For example, Pereira et al. [34] reported the ADC_mean_ value of PNETs was significantly higher in G1-PNETs, while Hwang et al. [19] indicated that ADC showed no significant difference between the G1 and G2 + 3 tumors. Second, variable MR scanners as well as different protocols were used in our study due to the retrospective multicenter design. As we all know, the equipment and setting of the protocols would have great influence on the results of ADC. Thirdly, the patients recruited were relative a small size, only 40 patients with pathological LN information and MR images were enrolled in the previous study [33]. Therefore, a prospective study with larger sample size these data should be conducted in the further.

Previous studies have shown the prognostic value of LNM with disease-free survival and overall survival in PNETs patients [10, 15, 20, 35, 36]. In this study, we applied LNM as a stratifying factor and evaluated the model from MRI features in prediction the patient outcome. The DFS in our group showed significant differences between the pathological LNM and non-LNM patients. Similar results were acquired with the independent MRI features-predicted LNM and negative patients, indicating the prognostic value of the MRI features in the management of PNETs. In other words, if nodal metastases were suggested by preoperative MRI features in NF-PNETs patients, resection with lymphadenectomy should be commonly performed. On the contrary, lymph node dissection was not advocated when the risk of LNM is very low.

This study has several limitations. First, as a retrospective multicenter study, the bias in patient selection and validation is inevitable, which may contribute to heterogeneity in the study. Second, variable MR scanners and protocols were used due to the retrospective multicenter design, although the morphological classification and measurement of short axis of the largest LN were not obvious affected by the different scanners and protocols. Thirdly, the proposed model showed relatively low sensitivity (76.2% in the training group and 75.0% in the validation group). In contrast, satisfactory specificity was obtained in both the training (93.3%) and validation group (88.1%), which means that this group of NF-PNETs may not need to receive the unnecessary lymphadenectomy and therefore avoid overtreatment for low-risk NF-PNETs patients. The cutoff value was selected using the maximum Youden’s index method which balanced sensitivity and specificity, because it can provide the most accurate LNM prediction for clinical decision-making. Factors for increasing sensitivity still need to be explored and integrated into model for LNM prediction, which will be focused in further studies. In addition, although irregular margin of tumor was considered as a predictor for LNM, the range of confident interval for OR was too broad (95% CI, 1.093–20.404) to need further studies to validate the results. Lastly, information of other MR sequences, such as DCE sequence, was not included and should be investigated in future studies.

In conclusion, we construct a simple and novel method to predict the LNM of NF-PNETs preoperatively based on irregular shape of primary tumor and short axis of the largest lymph node in regional area from MR images. As a noninvasive and steadily method, the MR predictors may serve as a biomarker to facilitate selection of optimal surgical approach and guide personalized treatment and surveillance in NF-PNETs patients.

## Supplementary Information

**Additional file 1**. MR protocols and scaning parameters.

## Data Availability

The datasets used and/or analyzed during the current study are available from the corresponding author on reasonable request.

## Abbreviations

ADC: Apparent diffusion coefficient
ALT: Alanine aminotransferase
AST: Aspartate aminotransferase
AUC: Area under curve
BMI: Body mass index
CA199: Carbohydrate antigen 199
CA724: Carbohydrate antigen 724
CBDD: Common bile duct dilatation
CEA: Carcinoembryonic antigen
CT: Computed tomography
DFS: Disease-free survival
DWI: Diffusion weighted imaging
EPI: Echo-planar imaging
FBG: Fasting blood glucose
FOV: Field of view
FSE: Fast spin echo
LN: Lymph node
LNM: Lymph node metastasis
MPDD: Main pancreatic ductal dilatation
MRI: Magnetic resonance imaging
NF‑PNETs: Nonfunctioning PNETs
NPV: Negative predictive value
NSE: Neuron-specific enolase
ORs: Odds ratios
OS: Overall survival
PNETs: Pancreatic neuroendocrine tumors
PPV: Positive predictive value
ROC: Receiver operating characteristic
TB: Total bilirubin
TE: Echo time
TR: Repetition time
