# Supplementary material for: Preoperative prediction of lymph node metastasis in nonfunctioning pancreatic neuroendocrine tumors from clinical and MRI features: a multicenter study
Source: Insights Imaging. 2022 Oct 8;13:162. doi: 10.1186/s13244-022-01301-9 (PMC9547759; doi:10.1186/s13244-022-01301-9)
Supplement: Supplementary file 1 — Additional file 1. MR protocols and scaning parameters. [file 13244_2022_1301_MOESM1_ESM.pdf]

ELECTRONIC SUPPLEMENTARY MATERIAL

Preoperative prediction of lymph node metastasis in nonfunctioning pancreatic neuroendocrine tumors from clinical and MRI features: a multicenter study

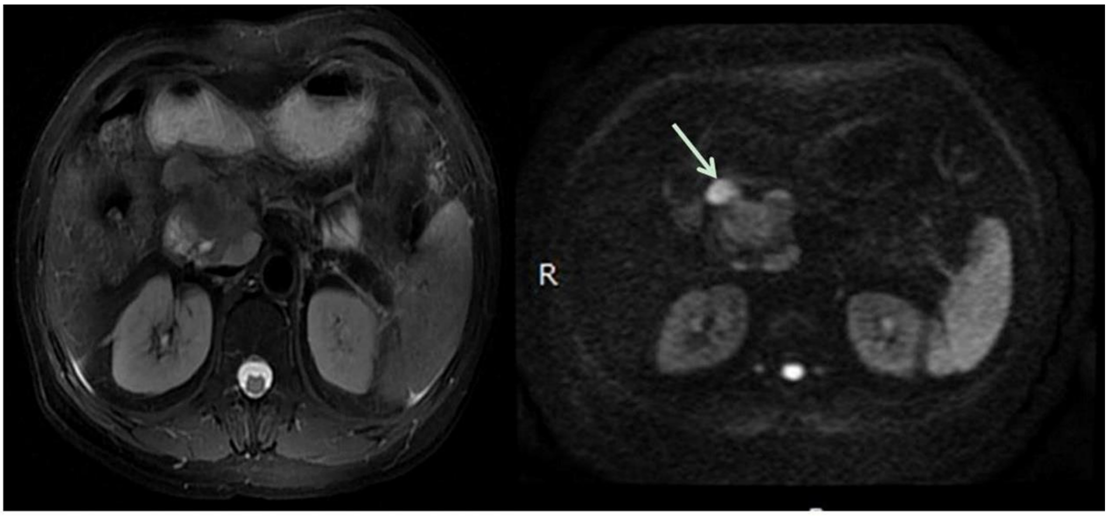

Fig. 1a Fig. 1 b

Fig S1. A 56-year-old woman with LNM in NF-PNETs. A, Axial FSE-T2 weighted image (T2WI) shows an irregular hyper-intense tumor (white arrow) in the head of the pancreas and an obvious enlarged lymph node was detected at anterior surface of the pancreatic head. B. The enlarged lymph node showed rim-like hyper-intense at DWI sequence (b=1000 1000 s/mm^2). The lymph node was proved as metastasis on pathology.

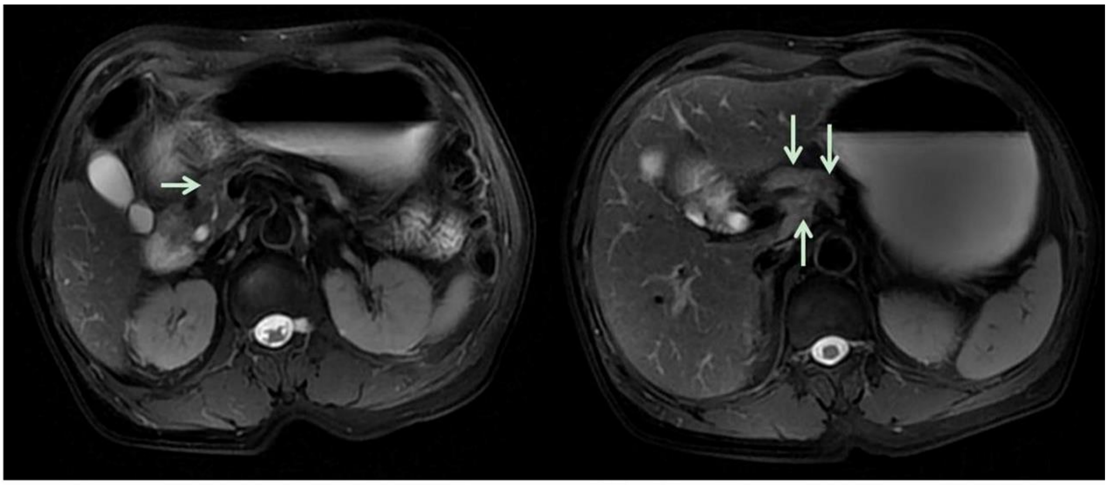

Fig. 2 a Fig. 2 b

Fig S2. A 65-year-old man without LNM in NF-PNETs. A, As shown on T2WI, a small round tumor was detected in the neck of the pancreas. B, Multiple enlarged lymph nodes were detected at hepatic hilar indicating the possible involvement. However, no metastatic lesions were found in the lymph nodes on histological examination.

Supplementary Table 1. MRI Parameters

	T1-weighted imaging			T2WI-weighted imaging			Diffusion--weighted imaging		
	1.5T (GE)	3.0T (GE)	1.5T (Siemens)	1.5T (GE)	3.0T (GE)	1.5T (Siemens)	1.5T (GE)	3.0T (GE)	1.5T (Siemens)
Sequences	Axial FSE T1WI			Axial FS-FSE T2WI			Axial SE-EPI		
TR/TE(msec)	4.2/min	3.2/2	6.7/2.4	10588/102	8000/109	2200/86	4500/min	6,000/93.3	4600/68
Flip angle	12°	15°	10°	110°	110°	140°	90°	90°	90°
Thickness/Gap (mm)	5/1	5/1	5/1	5/1	5/1	5/1	5/1	5/1	5/1
NEX	1	1	1	2	4	1	1-6	1-6	1-4
Matrix	224x192	256x192	320x240	320x320	288x256	320x320	140x96	128x128	140x140
